# *In vitro* adhesion, pilus expression, and *in vivo* amelioration of antibiotic-induced microbiota disturbance by *Bifidobacterium* spp. strains from fecal donors

**DOI:** 10.1080/19490976.2023.2229944

**Published:** 2023-07-04

**Authors:** Aki Ronkainen, Imran Khan, Eva Krzyżewska-Dudek, Kaisa Hiippala, Tobias L. Freitag, Reetta Satokari

**Affiliations:** a Human Microbiome Research Program, Faculty of Medicine, University of Helsinki, Helsinki, Finland; b Translational Immunology Research Program, Faculty of Medicine, University of Helsinki, Helsinki, Finland; c Department of Immunology of Infectious Diseases, Hirszfeld Institute of Immunology and Experimental Therapy, Polish Academy of Sciences, Wroclaw, Poland

**Keywords:** Bacteriotherapy, dysbiosis, fecal microbiota transplantation, gut microbiota, next-generation probiotics

## Abstract

Fecal microbiota transplantation (FMT) is used routinely to treat recurrent *Clostridioides difficile* infection (rCDI) and investigated as a treatment for numerous conditions associated with gut microbiota alterations. Metagenomic analyses have indicated that recipient colonization by donor bacteria may be associated with favorable clinical outcomes. Bifidobacteria are abundant gut commensals associated with health. We have previously demonstrated that *Bifidobacterium* strains transferred in FMT can colonize recipients in long term, at least for a year, and recovered such strains by cultivation. This study addressed *in vitro* adhesion and pilus gene expression of long-term colonizing *Bifidobacterium* strains from FMT donors as well as *in vivo* colonization and capability to ameliorate antibiotic-induced microbiota disturbance. RNA-Seq differential gene expression analysis showed that the strongly adherent *B. longum* strains DY_pv11 and DX_pv23 expressed tight adherence and sortase-dependent pilus genes, respectively. Two *B. longum* strains, adherent DX_pv23 and poorly adhering DX_pv18, were selected to address *in vivo* colonization and efficacy to restore antibiotic-disturbed microbiota in C57BL/6 murine model. DX_pv23 colonized mice transiently with a rate comparable to that of the *B. animalis* BB-12 used as a reference. Although long-term colonization was not observed with any of the three strains, 16S rRNA gene profiling revealed that oral administration of DX_pv23 enhanced the recovery of antibiotic-disturbed microbiota to the original configuration significantly better than the other strains. The findings suggest that selected strains from FMT donors, such as DX_pv23 in this study, may have therapeutic potential by *in vitro* expression of colonization factors and boosting endogenous gut microbiota.

## Introduction

Human gut microbiota is strongly associated with health. Individuals suffering from gut-related as well as systemic conditions, such as irritable bowel syndrome (IBS), inflammatory bowel disease (IBD), or metabolic syndrome, show altered (dysbiotic) gut microbiota when compared to healthy individuals.^1–3^ Dysbiosis can be defined as compositionally and functionally altered microbiota, usually manifesting itself as reduced species diversity, depletion of anaerobic commensal species and lower temporal stability as well as reduced production of short chain fatty acids. Dysbiosis underlines also compromised colonization resistance in recurrent *Clostridioides difficile* infection (rCDI).^3^ Fecal microbiota transplantation (FMT) is a medical procedure in which donor microbes are employed to resolve a recipient’s dysbiosis and alleviate a related medical condition. Currently, FMT is used routinely to treat rCDI whereas its suitability to treat other conditions is still inconclusive and under investigation.^2,3^ A recent metagenomic analysis of FMT-treated patients with various indications and donors suggests that higher donor strain colonization is associated with favorable clinical outcome.^4^ Cohorts from longitudinal FMT studies provide an excellent tool to track successfully transplanted bacterial taxa and pin-point commensals capable of long-term colonization. Commensal strains with strong colonization capacity could be employed in therapeutic purposes to treat dysbiosis-associated conditions and possibly achieve long-term health benefits.

*Bifidobacterium* spp. represent potential candidates for bacteriotherapy. Bifidobacteria are typical human commensals, and they are present in the gut throughout the course of human lifespan, albeit their relative abundance and species composition is subject to change over time.^5–7^ Bifidobacteria are considered to participate in various gut functions, such as nutrient processing and vitamin production, fostering of the host immune system, and pathogen prevention.^6–10^ Due to their observed health-associated properties, many bifidobacterial strains have a long history of use as probiotics.^11^ Bacterial colonization of the gastrointestinal tract is a multifaceted process that depends on many features, collectively designated as colonization factors.^6,7^ Ability to adhere to the intestinal epithelium is considered to facilitate colonization. Bifidobacteria have been described to mediate adhesion by pili, capsular and exopolysaccharides, teichoic acids, serine protease inhibitors, and other proteins, and thus far pili are the best described bifidobacterial adhesins with annotated genes for their biogenesis.^6–8,12^

Bifidobacteria have been reported to express at least two different pilus types: sortase-dependent pilus and type IVb tight adherence pilus (Tad pilus).^7,13^ A study by Milani et al.^9^ indicated that sortase-dependent pilus genes exhibit variation among bifidobacteria as they are not present in all strains and the number of encoding loci depends on the species or strain. The locus comprises a major pilin protein gene (*fimA*/*P*), one to two genes for ancillary pilin proteins (*fimB* and/or *fimQ*), and pilus-specific sortase gene (*strA*).^7,13^ Different sortase-dependent pili seem to interact with different structures, such as proteins of extracellular matrix and/or diet-derived glycans, suggesting that these pili may perform different ecological activities.^9^ Unlike sortase-dependent pilus, Tad pilus gene cluster is highly conserved among bifidobacteria.^7,13^ Based on their work with *B. breve* UCC2003, O’Connell Motherway et al.^14^ proposed that bifidobacterial *tad* cluster contains gene domains for pilus assembly and localization (*tadA*, *tadB*, *tadC*, and *tadZ*), pilin proteins (*flp*, *tadE*, and *tadF*), and processing (*tadV*, outside the *tad* locus). Tad pilus shows affinity for glycoproteins and glycolipids present on intestinal epithelial cells, underlying its role in bifidobacterial adhesion.^13^

Commercial probiotic bifidobacterial strains do not colonize adult human gut in long term, but FMT cohorts with rCDI patients have shown stable reestablishment of bifidobacterial populations, and *B. bifidum* and *B. longum* were found to be among the most-colonizing donor species.^4,11,15,16^ As FMT entails the transfer of a complex ecosystem from one person to another, colonization of donor bifidobacteria may be promoted by different factors, such as microbial collaboration and receptive host microbiota. Bifidobacterial species have been documented to interact with each other as well as other gut commensals, such as *Bacteroides* spp., *Eubacterium rectale*, and *Faecalibacterium prausnitzii* in mutualistic cross-feeding.^6^ Furthermore, rCDI patients usually have antibiotic-induced dysbiosis that may provide bifidobacteria with suitable niches, enabling colonization to begin with. Indeed, it has been demonstrated that antibiotic pre-treatment of mice enhances bifidobacterial colonization in the FMT context.^17^

Previously, we followed the transfer of bifidobacteria from FMT donors to rCDI patients.^16^ By combining molecular methods, cultivation, and whole-genome sequencing, we were able to follow donors´ bifidobacteria in the patients and verify that certain strains of *B. adolescentis*, *B. longum*, and *B. pseudocatenulatum* could colonize the patients even up to one year after FMT. The efficient colonization capacity makes the strains interesting candidates for bacteriotherapy in which long-lasting effect is of essence. In this study, our aim was to gain deeper insight on the strains’ adhesion and expression of colonization factors as well as their colonization capacity and potential to ameliorate antibiotic-induced dysbiosis in mice.

## Materials and methods

### Bacterial strains and culture conditions

Bifidobacterial strains used in the study are presented in Table 1. All the donor strains originate from FMT donors and have shown successful colonization in rCDI patients.^16^ As for the reference strains, *Bifidobacterium animalis* subsp. *lactis* BB-12® was obtained from a commercial product (Probiootti comp, Suomen Bioteekki, Finland) while the rest were obtained from the German Collection of Microorganisms and Cell Cultures GmbH (Leibniz Institute DSMZ, Germany). Bifidobacterial cultures were grown under anaerobic conditions at +37°C for 48 ± 4 h on Lactobacilli MRS agar or broth (Neogen Culture Media, CAT#NCM0035A/NCM0079A) supplemented with 0.5 gl^−1^ of L-cysteine (Sigma-Aldrich, CAT#30129) (hereinafter MRSc agar/broth).

**Table 1.** *Bifidobacterium* spp. strains used in the study.

Strain	Detected in rCDI patients	Used in this study
Donor strains*		
*B. adolescentis* DX_pv1	1-year post-FMT in 1 patient	adhesion, RNA-Seq, MALDI-TOF MS
*B. longum* DX_pv18	1-year post-FMT in 2 patients	adhesion, RNA-Seq, colonization, MALDI-TOF MS
*B. longum* DX_pv23	1-year post-FMT in 2 patients	adhesion, RNA-Seq, colonization, MALDI-TOF MS
*B. longum* DX_pv32	1-year post-FMT in 1 patient	adhesion, RNA-Seq, MALDI-TOF MS
*B. longum* DY_pv11	1-year post-FMT in 3 patients	adhesion, RNA-Seq, MALDI-TOF MS
*B. pseudocatenulatum* DX_pv5	4-months post-FMT in 2 patients	adhesion, RNA-Seq, MALDI-TOF MS
**Reference strains**		
*B. adolescentis* DSM 20,083	n/a	MALDI-TOF MS
*B. angulatum* DSM 20,098	n/a	MALDI-TOF MS
*B. animalis* subsp. *lactis* BB-12	n/a	adhesion, colonization, MALDI-TOF MS
*B. animalis* subsp. *lactis* DSM 10,140	n/a	MALDI-TOF MS
*B. bifidum* DSM 20,456	n/a	MALDI-TOF MS
*B. breve* DSM 20,213	n/a	MALDI-TOF MS
*B. catenulatum* DSM 16,992	n/a	MALDI-TOF MS
*B. dentium* DSM 20,436	n/a	MALDI-TOF MS
*B. gallicum* DSM 20,093	n/a	MALDI-TOF MS
*B. longum* subsp. *infantis* DSM 20,088	n/a	MALDI-TOF MS
*B. longum* subsp. *longum* DSM 20,219	n/a	MALDI-TOF MS
*B. pseudocatenulatum* DSM 20,438	n/a	MALDI-TOF MS

*Isolation described by Jouhten et al. 2020.^16^

Selective cultivation of bifidobacteria from mouse feces was done on MRSc agar supplemented with 0.05 gl^−1^ of mupirocin (Sigma-Aldrich, µ CAT#69732) (hereinafter MUP agar).^16,18^ Prior to cultivation, fecal pellets were suspended 1:10 (w/v) in phosphate-buffered peptone water (Sigma-Aldrich, CAT#77187) supplemented with 0.5 gl^−1^ of L-cysteine and diluted further with the same solvent. Dilutions were spread on MUP agar plates and grown as above.

### Adhesion experiments

Donor strains (Table 1) were examined for adhesion to intestinal mucus by the method described by Kainulainen et al.^19,20^ In detail, wells of a MaxiSorp™ microtiter plate (Thermo Scientific, CAT#445101) were coated with 75 ng of porcine mucus (Sigma-Aldrich, CAT#M2378) by overnight incubation at +4°C and washed three times with 200 µl of 1X PBS (pH 7.4). For each strain, parallel inoculations from the same colony were made into MRSc broth supplemented with and without 10 µlml^−1^ of tritiated thymidine (17,6 Ci mmol^−1^, PerkinElmer, CAT#NET355005MC) with the non-supplemented culture preserved for RNA extraction (see below). After growing (see above), cells were harvested by centrifugation, washed with and resuspended in PBS, and adjusted to optical density (OD_600_) of 0.250. One hundred microliters of cell suspension was pipetted on mucus and incubated at +37°C for 1 h. Another 100 µl aliquot was pipetted into Optisafe HiSafe™ 3 liquid scintillation cocktail (PerkinElmer, CAT#1200.437) to serve as a reference for the cells added to the well. The wells were washed three times with PBS to remove non-adherent cells and treated with 1% SDS − 0.1 M NaOH at +37°C overnight to lyse adherent cells. After pipetting cell lysates into liquid scintillation cocktail, the radioactivity of lysates and corresponding references were measured with the Wallac Winspectral 1414 liquid scintillation counter (PerkinElmer). Experiments were done in three biological replicates each including three technical replicates with the reference strain *B. animalis* BB-12 (Table 1) serving as a positive control. For each experiment, strain-specific adhesion (%) was determined from the ratio between the radioactivity emitted by adhered cells and added cells by using arithmetic means of respective technical replicates (± standard deviation).

### RNA extraction and transcriptome sequencing

Donor strain cultures from three biological replicates of adhesion experiments (see above) were preserved for RNA extraction to perform gene expression analysis by RNA-seq. Cells were harvested by centrifugation, resuspended in RNA*later*® (Thermo Fisher, CAT#AM7020), kept at +4°C overnight, and stored at −20°C until extraction. Thawed samples were mixed 1:9 (v/v) with MetaPolyzyme (Sigma-Aldrich, CAT#MAC4L), incubated at +35°C for 90 min, and centrifuged at 5000 g for 5 min with supernatant being replaced with RLT lysis buffer (RNeasy Minikit, Qiagen, CAT#74104) supplemented with 10 µlml^−1^ β-mercaptoethanol (Sigma-Aldrich, CAT#M6250). Resulting mixtures were transferred into tubes containing 200–250 mg of acid-washed glass beads with diameter of 212–300 µm (Sigma-Aldrich, CAT#G1277) pretreated in similarly supplemented RLT lysis buffer overnight. The tubes were treated with the Fastprep®-24 instrument (MP Biomedicals) at 5.5 ms^−1^ for 60 s. RNA was extracted from the homogenates by RNeasy Minikit according to the manufacturer’s instructions, including the optional DNase I treatment. RNA concentration and purity were determined with the NanoDrop™ ND-1000 spectrophotometer (Thermo Scientific).

The samples were sent for RNA integrity number (RIN) assignment and subsequent RNA-Seq to the Biomedicum Functional Genomics Unit (FuGU) at the University of Helsinki (Finland). At FuGU, RIN assignment was done with RNA ScreenTape® (Agilent Technologies). One thousand nanograms of total RNA was ribo-depleted using NEBNext® rRNA Depletion Kit (New England Biolabs, CAT#E7850). Library preparation was completed on ribo-depleted RNA using the NEBNext® Ultra™ Directional II RNA Library prep kit (New England Biolabs, CAT#E7760) using 8 cycles of PCR amplification and indexed using unique dual indexing. Indexed library preps from each sample were then pooled and sequenced with 75SE reads at a pool concentration of 1.6 pM on the NextSeq 500 using a NextSeq High Output 75 cycle flow cell (Illumina). Illumina’s BCL to FASTQ file converter (bcl2fastq v2.20.0.422) was used to convert BCL files to FASTQ file format and demultiplex the samples.

### Differential gene expression analysis

For transcriptomic analysis, we generated raw Illumina sequencing fastq reads from six strains with three replicates per strain. The read quality control was performed using fastp and checked with FastQC and MultiQC.^21–23^ The strain-specific genomes were retrieved from the European Nucleotide Archive with the four *B. longum* strains used for pangenome construction using Roary.^16,24^ The reads were aligned to the respective reference genome and the resulting read counts were used to estimate differentially expressed genes (DEGs) with edgeR.^25–27^ The pairwise transcriptome comparisons of DEGs were performed with an adjusted p-value threshold of <0.5 and absolute log2 (fold change) value of >1.

### Mouse experiment

**Figure 1.**
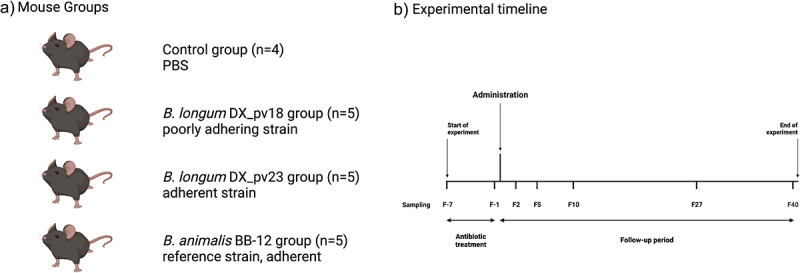
Design of the colonization experiment in mice.

Based on the results obtained from the adhesion experiments, we designed an experiment in mice to address the colonization capacity of selected bacterial strains. We selected two strains that originated from the same FMT donor and were of the same species but differed in their adhesion to mucus. The strains, poorly adhering *B. longum* DX_pv18 and adherent *B. longum* DX_pv23, were tested in parallel for their ability to colonize the intestine and to ameliorate antibiotic-induced dysbiosis as compared to the reference strain *B. animalis* BB-12 (Figure 1).

(a) Four groups of mice (*n* = 4–5) were administered by oral gavage with a bifidobacterial strains or PBS (control): poorly adhering *B. longum* DX_pv18, adherent *B. longum* DX_pv23, and adherent reference *B. animalis* BB-12. (b) All the groups were treated with antibiotics for seven days before administration of bacterial strains. Sampling of fecal pellets was performed before and after antibiotic treatment (F-7 and F-1, respectively) as well as 2, 5, 10, 27, and 40 days after administration (F2, F5, F10, F27, and F40, respectively). Mice were sacrificed 56 days post-administration.

Adult C57BL/6 mice were originally purchased from The Jackson Laboratory (Bar Harbor, Maine), maintained under specific pathogen-free conditions in ventilated cages and fed with sterilized chow (Envigo, CAT#2016C). Filtered tap water was provided without restriction, and cages were cleaned once a week. The microbial status of the animal facility was regularly monitored by performing annual tests for FELASA-listed pathogens as well as quarterly serological and bacteriological tests. Breeders produced 7 litters from which 18 female offspring were mixed into four groups so that each group had mice from at least 4 different litters. This resulted in three groups of five mice each (groups that received bifidobacteria) and control group of three mice. The control group was supplemented with one retired female breeder to increase the group size. All the groups were maintained for 1 week, after which they were treated for seven days with antibiotic cocktail dissolved in drinking water. The cocktail consisted of 660 mgl^−1^ of ciprofloxacin (Tamro, Finland), 1 gl^−1^ of metronidazole and 500 mgl^−1^ of vancomycin (both from Yliopiston Apteekki, Finland). One day after the treatment, bifidobacterial groups were administered by oral gavage with 10^9^ CFU of either *B. longum* DX_pv18, *B. longum* DX_pv23, or *B. animalis* BB-12 suspended in 200 ml of sterile PBS. The control group was administered with an equal volume of PBS. The study design is summarized in Figure 1a.

Fecal pellets for selective cultivation and sequencing-based microbiota analysis (see below) were collected before starting the antibiotic treatment, on the last day of the antibiotic treatment, as well as on days 2, 5, 10, 27, and 40 after the administration of bifidobacteria (Figure 1b). Prior to every sampling, mice were transferred to separate autoclaved cages for 2 h to obtain samples from individual animals and provided with food and water as above. Mice were weighed before returning them to the group cage. Mice were euthanized 56 days post-administration. Colon weight (full and empty) and colon length (empty) were measured. All the procedures involving animals were approved by the Southern Finnish State Administrative Agency (ESAVI/11326/2020).

### Selective cultivation and identification of bifidobacteria

After every sampling, fecal pellets were homogenized with a 10 mg aliquot from each sample taken immediately to selective cultivation (see above). The remaining sample was stored at −80°C for DNA extraction (see below). MUP agar plates yielding 20–200 CFU were screened for different colony types that were cultivated on MRSc agar. Resulting pure cultures were examined for colony morphology, Gram reaction, and cell morphology. Isolates meeting the screening criteria for bifidobacteria (i.e., Gram-positive cells with *Bifidobacterium*-like morphology) were identified by MALDI-TOF MS at Vita Laboratories, Inc. (Finland). The ability of MALDI-TOF MS to distinguish and identify *Bifidobacterium* spp. was first verified by using the 11 reference strains obtained from DSMZ as well as *B. animalis* BB-12 (Table 1, Supplementary Table S1).

### DNA extraction and 16S rRNA gene amplicon sequencing

DNA extraction from fecal pellets was done by the method described earlier.^28^ Accordingly, 20 mg samples were pretreated by repeated bead beating and subjected to high-throughput DNA extraction by KingFisher™ Flex 96 (Thermo Fisher Scientific). The extraction included three blank samples to assess potential contamination. DNA concentration was determined with Quant-iT™ PicoGreen™ dsDNA Assay Kit (Invitrogen™, CAT#P7589). ZymoBIOMICS Microbial Community DNA Standard (Zymi Research, CAT#D6306) was used as a sequencing control.

The samples were sent for 16S rRNA gene amplicon sequencing to the Institute of Biotechnology (BI) at the University of Helsinki (Finland). At BI, the V3-V4 regions of the 16S rRNA gene were amplified by a previously published protocol with the following changes:^29^ two technical replicates (25 μl reactions) per sample, and a mixture of the universal bacterial primers 341F1–4 (5′ CCTACGGGNGGCWGCAG 3′) and 785R1–4 (5′ GACTACHVGGGTATCTAATCC 3′) with partial Illumina TruSeq adapter sequences added to the 5′ ends (F1; ATCTACACTCTTTCCCTACACGACGCTCTTCCGATCT, F2; ATCTACACTCTTTCCCTACAC GACGCTCTTCCGATCTgt, F3; ATCTACACT CTTTCCCTACACGACGCTCTTCCGATCTagag, F4; ATCTACACTCTTTCCCTACACGACGCTC TTCCGATCTtagtgt and R1; GTGACT GGAGTTCAGACGTGTGCTCTTCCGATCT, R2; GTGACTGGAGTTCAGACGTGTGCTCTT CCGATCTa, R3; GTGACTGGAGTTC AGACGTGTGCTCTTCCGATCTtct, R4; GT GACTGGAGTTCAGACGTGTGCTCTTCCGATCTctgagtg). Additional nucleotides (lowercase letters) were introduced for mixing in sequencing. The two-step PCR and subsequent quantification, pooling, and purification were done as previously.^29^ The PCR amplicon pool was checked using Fragment Analyzer (Advanced Analytical Technologies Inc.). All PCR batches included a blank sample (no added DNA template) to assess potential contamination. Finally, the PCR products were sequenced with Illumina MiSeq (v3 600 cycle kit) with 325 bases for the forward and 285 bases for the reverse read.

### 16S rRNA gene amplicon data analysis

The quality control of raw paired-end fastq sequence reads was performed using FastQC and MultiQC.^22,23^ PCR primers were removed using Cutadapt and the reads were analyzed with dada2 pipeline.^30,31^ SILVA database release 138 was used for the taxonomy assignment of amplicon sequence variants (ASVs).^32^ Spurious and low abundance/prevalence reads as well as unclassified ASVs were filtered at phylum level and ASVs belonging to Archaea, Chloroplast, Mitochondria and Euryarchaeota were removed.

The data analyses were carried out using phyloseq R package.^33^ The microbiota count data was transformed to account for differences in library size, variance, scale, etc. For alpha diversity measures, we calculated Chao1 richness estimate and Shannon diversity index. The comparisons were done using pairwise Wilcoxon test with false discovery rate (FDR) correction using the Benjamini – Hochberg procedure. For beta diversity, rarefied counts were used for ordination analyses by binary Jaccard distance with non-parametric multidimensional scaling (NMDS).^34^ The PERMANOVA (pairwise adonis, 999 permutations) with binary Jaccard distance was used to compare diversity between the groups at the last time point (F27). The homogeneity level of within-group dispersion was checked using the betadisper function in vegan R package.^35,36^ We also observed differences in the relative abundance of major phyla across time points as well as presence of selected species.^34^

### Statistical analysis

Concerning the mouse parameters, all graphs were prepared, and data analyzed with GraphPad Prism (version 9.2.0 for Windows, GraphPad Software). Data normality distribution was tested using Shapiro–Wilk test. ANOVA followed by Tukey’s multiple comparisons test was used for evaluating the statistical significance of the analyzed data. Statistical analysis concerning the sequencing data is described in the corresponding method sections.

### Ethical considerations

The bifidobacterial donor strains were originally isolated from healthy adult volunteers screened for eligibility as fecal donors participating in a clinical study, which was approved by the Ethics Committee of Hospital District of Helsinki and Uusimaa Finland (DnroHUS124/13/03/01/11).^16,37^ The study subjects provided informed consent. The animal study was reviewed and approved by the Southern Finnish State Administrative Agency (ESAVI/11326/2020).

### Data availability

The sequencing data (16S rRNA gene amplicon and RNA-Seq) was deposited to the NCBI SRA database under the accession number PRJNA930167.

## Results

### *In vitro* adhesion of bifidobacterial strains

**Figure 2.**
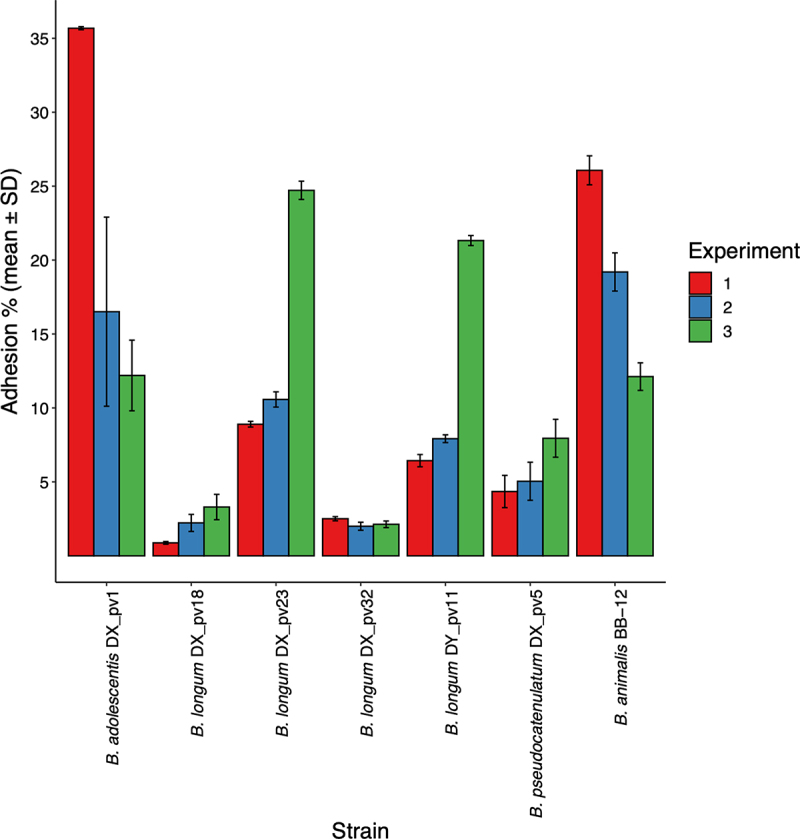
Adhesion of *Bifidobacterium* spp. strains to intestinal mucus.

First, we assessed the ability of six bifidobacterial donor strains to adhere to intestinal mucus *in vitro*. Adhesion was shown to be strain-dependent (Figure 2): *B. longum* strains DX_pv18 and DX_pv32 did not show appreciable adhesion and were considered poorly adhering (adhesion level in average <3%), whereas *B. pseudocatenulatum* DX_pv5 showed moderate adherence (in average 6%). *B. adolescentis* DX_pv1 as well as *B. longum* strains DX_pv23 and DY_pv11 showed high adherence (in average >12%). *B. animalis* BB-12 was highly adherent as expected for a positive control.^38^

Data is presented from three biological replicates (experiments 1–3) as percentual means of adhered bacteria from that of added bacteria (adhesion %). Error bars represent standard deviations of three technical replicates included in each experiment. *B. animalis* BB-12 was used as a positive control.

### Expression of pilus genes

**Figure 3.**
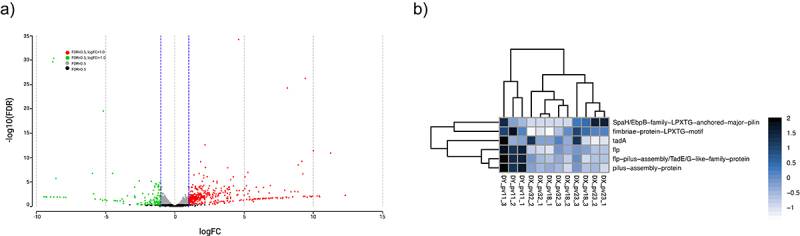
Differential gene expression among *B. longum* strains.

To obtain insight into the expression of potential adhesins possessed by the strains, we used RNA-seq to explore gene expression during the adhesion experiments. Gene expression comparison of adherent *B. longum* strains DX_pv23 and DY_pv11 to that of poorly adhering *B. longum* strains DX_pv18 and DX_pv32 revealed 536 differential expressed genes (Figure 3a). There were 406 upregulated and 130 downregulated genes among the adherent strains (adjusted p-value (FDR) threshold of <0.5 and absolute log2 (fold change) value of >1) (Figure 3a). The comparison showed six differentially expressed pilus genes of which four belonged to Tad pilus cluster and two to sortase-dependent pilus cluster (Figure 3b). Tad pilus genes were expressed by DY_pv11 whereas sortase-dependent pilus genes by DY_pv11 as well as DX_pv23. Interestingly, DX_pv23 also showed expression of Tad pilus genes in one out the three cultures. Overall, comparison between the adherent and poorly adhering *B. longum* strains showed that stronger *in vitro* adhesion to intestinal mucus was associated with the higher level of pilus gene expression. In addition, the variation in a strain’s expression levels between the three biological replicates (Figure 3b) reflected the strain’s inter-experiment adhesion levels (Figure 2). Concerning the strains of other species, *B. adolescentis* DX_pv1 (adherent) and *B. pseudocatenulatum* DX_pv5 (moderately adherent), RNA-seq analysis showed expression of genes for the Tad pilus cluster (Supplementary Table S2).

(a) Volcano plot showing differentially expressed genes (DEGs) by adherent *B. longum* strains DX_pv23 and DY_pv11 in comparison to that of poorly adhering *B. longum* strains DX_pv18 and DX_pv32. (b) Heatmap representing upregulated and/or downregulated pilus genes by the same strains. Numbers 1–3 following strain codes denote biological replicates corresponding to those in Figure 2.

### *In vivo* colonization and amelioration of antibiotic-induced dysbiosis by bifidobacterial strains

The mouse experiment was designed to assess colonization capacity and microbiota restoring effect of the poorly adhering *B. longum* DX_pv18 and adherent *B. longum* DX_pv23. The adherent probiotic *B. animalis* BB-12 was used as a comparative reference along with the carrier solution PBS as a negative control.

First, we followed the colonization of orally administered strains by selective cultivation of mouse feces. Bacterial isolates were examined for colony morphology, Gram reaction, and cell morphology. Among the isolates (*n* = 540), there were seven different colony types comprising four different Gram-positive cell morphologies (Suplementary Table S1). Five out of seven colony types on MUP agar were present in all mouse groups at all time points, representing endogenous bacteria of the mouse strain. The remaining two colony types were different from the endogenous colonies and appeared like those of administered strains *B. longum* DX_pv23 and *B. animalis* BB-12. Several isolates of each colony type (*n* = 120) were identified with MALDI-TOF MS, which was first verified for the ability to distinguish and identify *Bifidobacterium* spp. by analyzing the reference strains (Table 1). MALDI-TOF MS identified three endogenous colony types with identical cell morphology as *Ligilactobacillus murinus* while the other two colony types with the same cell morphology were identified as *B. pseudolongum*. Thus, according to selective cultivation with MUP agar, the only endogenous representative of the genus *Bifidobacterium* in mice was *B. pseudolongum*. This species was detected in all mouse groups throughout the experiment (Table 2). As for the administered strains, the adherent *B. longum* DX_pv23 was detected in two mice two days after administration. The strain was identified by the rep-PCR method used earlier in the study by Jouhten et al.,^16^ as MALDI-TOF MS was not able to identify the strain. *B. animalis* BB-12 was detected in two mice two days after administration and in one mouse five days after administration (Table 2). The poorly adhering strain *B. longum* DX_pv18 was not detected at any time point.

**Table 2.** Presence of *Bifidobacterium* spp. in mouse feces by selective cultivation.

Mouse group	Time point^1^ and number of mice with confirmed presence of strain^2^									
	F-7		F-1		F2		F5		F10	
	Bp	AS	Bp	AS	Bp	AS	Bp	AS	Bp	AS
Control (*n* = 4)	4	-	4	-	4	-	4	-	4	-
*B. longum* DX_pv18 (*n* = 5)	5	-	5	-	5	-	5	-	5	-
*B. longum* DX_pv23 (*n* = 5)	5	-	5	-	5	2	5	-	5	-
*B. animalis* BB-12 (*n* = 5)	5	-	5	-	5	2	5	1	5	-

^1^F-7 and F-1: before and after antibiotic treatment, respectively; F2, F5, F10: 2, 5, and 10 days after administration.

^2^Bp: *B. pseudolongum*; AS: administrated strain.

**Figure 4.**
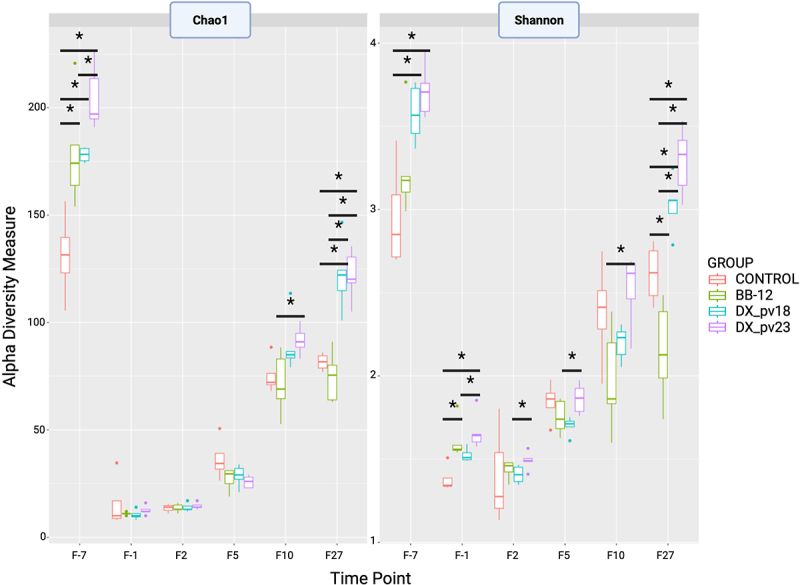
Bacterial richness and diversity in different treatment groups across time points.

Next, we looked at 16S rRNA gene amplicon sequencing data to evaluate alpha diversity in the mouse feces. The Microbial Community DNA Standard used as sequencing control passed quality control. Chao1 and Shannon indices were used as measures for bacterial richness and diversity, respectively (Figure 4). At the baseline, the bifidobacterial treatment groups differed from the control group in richness before the antibiotic treatment (Chao1), but after the treatment all the groups had equally low richness. After administration, the richness started to increase in all the groups, but the fastest recovery was observed in the DX_pv23 group. Ten days post-administration, the DX_pv23 group differed from the other groups with increased richness and 27 days post-administration the DX_pv23 and DX_pv18 groups differed from the BB-12 and control groups with higher richness. Diversity (Shannon) followed similar patterns as richness, albeit the groups were dissimilar in diversity after the antibiotic treatment. As for the mice, there was no difference in body weight between the groups during the experiment. Also, at the end of the experiment, the groups did not differ from each other in body weight (Supplementary Figure S1) nor in colon weight and length (Supplementary Figure S2).

Alpha diversity measures Chao1 richness estimate and Shannon diversity index. Comparisons between the intervention groups were done using pairwise Wilcoxon test with False Discovery Rate (FDR) correction using Benjamini–Hochberg procedure. Time points: F-7 and F-1: before and after antibiotic treatment, respectively; F2, F5, F10, F27: 2, 5, 10, and 27 days after administration. * *p* < 0.05.

**Figure 5.**
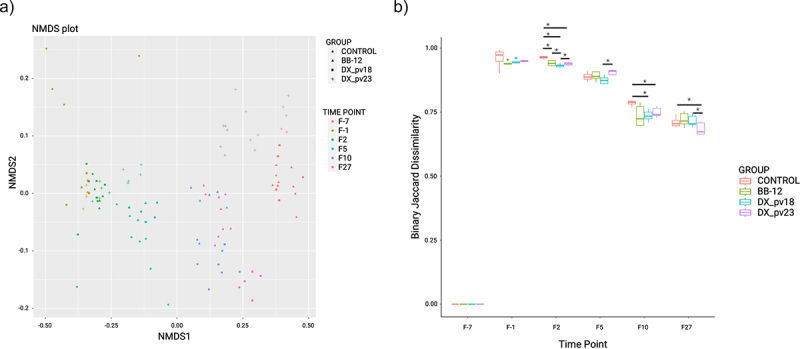
Bacterial beta diversity in different treatment groups across time points.

The similarity of overall microbiota composition was evaluated by non-metric multidimensional scaling (NDMS) plot based on binary Jaccard distance at the ASV level (Figure 5a). Post-antibiotic treatment samples from all the groups clustered near each other, after which the groups started to follow specific trajectories during the microbiota recovery. The PERMANOVA pairwise adonis test based on binary Jaccard permutation distances showed that the four groups were significantly different 27 days post-administration with FDR-corrected with Benjamini–Hochberg method (*P* < 0.05) with permutation test for homogeneity of multivariate dispersions being non-significant. The DX_pv23 group showed the fastest microbiota recovery from antibiotic disturbance as compared to the other groups. Twenty-seven days post-administration, the DX_pv23 group samples were nearest to the corresponding pre-antibiotic treatment samples, indicating the most complete recovery of microbiota.

(a) Non-parametric multidimensional scaling (NMDS) with binary Jaccard distance. (b) Boxplots of the beta diversity (calculated as Binary Jaccard dissimilarity) between samples grouped by time point. The Wilcoxon test was used to determine significant changes between groups at each time point. Time points: F-7 and F-1: before and after antibiotic treatment, respectively; F2, F5, F10, F27: 2, 5, 10, and 27 days after administration. * *p* < 0.05.

The comparison of binary Jaccard distance was used also as a measure for similarity between each time point and the baseline (shorter the distance, more similar the group) with the Wilcoxon test used to see significant differences between the groups (Figure 5b). The comparison confirmed the fastest and most complete microbiota recovery in the DX_pv23 group. As shown in Figure 5b, microbial communities at the latest time points had reverted toward the baseline with the DX_pv23 group being the most baseline-like. Furthermore, DX_pv18 seems to be more effective than the reference strain BB-12 in ameliorating antibiotic-induced microbiota disturbance (Figures 4 and 5).

**Figure 6.**
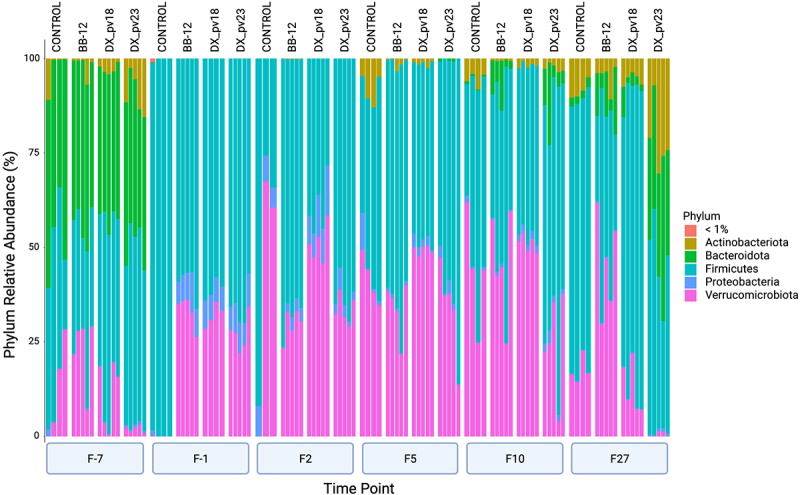
Bacterial composition of fecal mouse microbiota at phylum level for different groups and time points.

In assessing microbiota composition at bacterial phylum level, we observed that after antibiotic treatment, the relative abundance of two major phyla, Actinobacteriota and Bacteroidota, decreased in all the groups while there was an increase in the relative abundance of Proteobacteria (Figure 6). During the follow-up period, bacterial composition started to shift toward the pre-antibiotic state. Based on the major phyla, microbiota recovery was again fastest in the DX_pv23 group as evident by the increase in the relative abundance of the phyla Actinobacteriota and Bacteroidota 27 days post-administration. At the level of individual species, the endogenous bifidobacterial species *B. pseudolongum* was detected to some degree in all the groups before antibiotic treatment but not at the end of it and again five days after administration in all the groups except for DX_pv23 (Supplementary Figure S3). However, 27 days post-administration it was detected in all the groups with the highest read counts detected in the DX_pv23 group. Species of the administered strains, *B. animalis* and *B. longum* were detected only in the respective treatment groups (Supplementary Figure S3). Interestingly, gut commensal *Akkermansia muciniphila* increased in relative abundance after antibiotic treatment in all the groups except for the control but returned to the pre-treatment-level 27 days post-administration only in the DX_pv18 and DX_pv23 groups (Supplementary Figure S3).

Bars represent relative abundance of phyla accounting for 99% of sequences. Time points: F-7 and F-1: before and after antibiotic treatment, respectively; F2, F5, F10, F27: 2, 5, 10, and 27 days after administration.

## Discussion

The success of FMT in treating rCDI has clearly demonstrated how manipulation of gut microbiota may resolve a dysbiosis-associated medical condition.^2,3^ Recent studies have linked the clinical success of FMT to stable engraftment of donor bacteria.^4,39^ In this respect, two *Bifidobacterium* species, *B. bifidum* and *B. longum*, were found to be among the most successfully engrafted species.^4^ FMT may, despite its safety, involve risks yet unknown as it relies on transfer of complex ecosystem from one person to another, and thus there is a great interest in bacteriotherapy in which thoroughly characterized human commensals are employed to correct dysbiosis. Here, our aim was to study colonization and therapeutic potential of bifidobacterial strains isolated from FMT donors. The strains have previously shown successful colonization in rCDI patients in the FMT context.^16^ The strains are representatives of species *B. adolescentis*, *B. longum*, and *B. pseudocatenulatum*, the three most prevalent bifidobacterial species in healthy adult human gut, making them excellent candidates for therapeutic purposes.^40^

At first, we studied whether the strains’ capability for long-term colonization is associated with efficient adhesion to intestinal mucus. However, the strains differed in *in vitro* adhesion with *B. longum* strains DX_pv18 and DX_pv32 being poorly adherent, *B. pseudocatenulatum* DX_pv5 being moderately adherent, and *B. adolescentis* DX_pv1 as well as *B. longum* strains DX_pv23 and DY_pv11 being highly adherent. As two strains capable of long-term colonization showed poor adherence, it is possible that they require gut environment to express adhesins, or their adhesion mechanism is not related plainly to mucus, or they rely on different properties such as efficient nutrient scavenge and replication to reside in the human gut. As for the adherent strains, *in vitro* adhesion could be related to the expression of pilus genes.

Transcriptomic profiling in parallel with adhesion experiments showed that the strains differed in the expression of pilus genes. Adherent *B. longum* DY_pv11 expressed gene encoding pilus assembly protein TadE, a pseudopilin that decorates the pilin shaft formed by Flp as well as other pilus-related genes. In contrast, adherent *B. longum* DX_pv23 expressed mainly SpaH/EbpB family LPXTG-anchored major pilin, a type of sortase-dependent pilus, but in one out of the three experiments showed also the expression of Tad pilus related genes. Thus, adherent *B. longum* strains supposedly mediate their adhesion by different types of pili and may have a preference in the type of pilus they express *in vitro*. The poorly adhering *B. longum* strains showed significantly lower expression level for pilus genes of either type. Interestingly, the gene encoding TadE, has previously been reported to be expressed constitutively only *in vivo* in *B. breve* UCC2003.^14^ A study with *B. longum* FGSZY16M3 showed expression of Tad pilus genes except for *tadE* during biofilm formation.^41^ To our knowledge, *B. longum* DY_pv11 is the first bifidobacterial strain to show *tadE* expression *in vitro*. Also, *B. adolescentis* DX_pv1 and *B. pseudocatenulatum* DX_pv5 showed expression of Tad pilus genes based on mapped read counts, although in the lack of same species reference in this study we were not able to do a comprehensive DEG analysis. Considering that the *in vitro* pilus expression could be linked to the adhesion levels of the studied strains, pilus expression may be considered an advantageous property when considering potential probiotic use of the strains as it could contribute to their persistence in the human gut, as shown previously for *Lacticaseibacillus rhamnosus* GG.^42^

Finally, we assessed the colonization capacity of two *B. longum* strains in mice after oral administration as well as their efficacy in ameliorating antibiotic-induced disturbance of microbiota. For this trial, we chose *B. longum* strains that were originally isolated from the same FMT donor and showing long-term colonization in rCDI patients but differing in their adherence to mucus: the poorly adhering DX_pv18 and highly adherent DX_pv23. This approach enabled us to observe the strains’ colonization patterns after *in vitro* cultivation and in relation to their adhesive properties.

Selective cultivation on MUP agar allowed good assessment of bifidobacterial populations in mice, as previously described for human feces, although the medium also supported the growth of endogenous murine lactobacilli, especially *Ligilactobacillus murinus*. ^16,18^ Based on the cultivation, the colonization capacity of the administered strains reflected *in vitro* adhesion: the poorly adhering DX_pv18 was not detected in mouse feces after administration while the highly adherent DX_pv23 was detected two days post-administration. The highly adherent reference strain *B. animalis* BB-12 was detectable until five days post-administration. Thus, the adherent strains colonized mouse gut transiently. Results from the 16S rRNA gene sequencing analysis confirmed the cultivation results: *B. longum* and *B. animalis* were detected only in mice receiving the respective strain. DX_pv23 and BB-12 were detected in the same samples both by 16S rRNA gene sequencing and cultivation, while DX_pv18 was detected by sequencing only.

Our strains from FMT donors colonized mouse gut only transiently after oral delivery, although they were found to colonize rCDI patients in long term in the FMT context.^16^ In addition to differences in the administration mode, cross-species incompatibility is a likely explanation for the results. Previously, Grimm et al.^43^ followed the colonization of *B. bifidum* S17/pMGC, a strain of human origin, in C57BL/6J mice under specific pathogen-free and germ-free conditions and showed that the strain colonized mice only under the latter conditions. This observation suggests that germ-free conditions provided the *B. bifidum* strain of human origin with a suitable niche while pathogen-free conditions prevented colonization due to presence of endogenous mouse microbiota. In a similar fashion in the FMT context, Lundberg et al.^44^ showed that bifidobacteria present in fecal transplant of human origin could not colonize C57BL/6 mice and that human microbiota is a weak stimulator for the murine immune system. These studies underline the host-specificity of bifidobacteria, and demonstrate the limitations of murine models, in particular C57BL/6, in addressing the colonization capacity of bifidobacteria of human origin. Furthermore, a study with human subjects showed that the persistence of administered *B. longum* AH1206 was higher in individuals when endogenous strains of *B. longum* were underrepresented.^45^ Thus, colonization was influenced by a niche opportunity provided by the gut ecosystem. Concerning the current study, the mice harbored an endogenous species *B. pseudolongum* which may have affected the colonization of administered strains. The true colonization capacity of our strains following oral administration remains to be determined in further studies. As bifidobacteria hold the “generally regarded as safe” status, a study with human subjects is a feasible option for this purpose.

We used 16S rRNA gene high-throughput profiling to follow microbiota changes during the antibiotic treatment and the restoration period. Before the antibiotic treatment, the control group differed from the other groups with lower bacterial richness and diversity. It is plausible, that the inclusion of the older female breeder affected the result as indicated by the within-group variation (Figure 4). This is supported by the observation that the bacterial composition of the retired breeder mouse differed from the other mice in the group at the phylum level as the mouse lacked Verrucomicrobiota (Figure 6, F-7, control, column one). As expected, the antibiotic treatment affected the gut microbiota in all the groups by decreasing the richness and diversity drastically, particularly affecting the relative abundance of Actinobacteriota and Bacteroidota while increasing Proteobacteria, which is in line with a previous study using mice of the same strain and antibiotic cocktail.^17^ The control group differed from the other groups in terms of bacterial composition after the antibiotic treatment by the lack of Verrucomicrobiota, resembling in this respect the pre-antibiotic state of the retired breeder. It may be hypothesized that this resulted in part from the cage effect^46^ as the mice in each group were co-housed during the experiment. Nevertheless, according to the analyses, *B. longum* DX_pv23 appeared to promote the fastest recovery of microbiota during the follow-up period.

Although *B. longum* strains DX_pv18 and DX_pv23 colonized mice only transiently, their administration in a single dose contributed to microbiota recovery after antibiotic treatment. Moreover, the effect of both strains was superior to the probiotic strain *B. animalis* BB-12. The restorative effect was evident in the strains’ ability speed up restoration of richness and diversity, to boost endogenous bacteria, especially Actinobacteriota and Bacteroidota, and to achieve higher similarity to the pre-treatment microbiota as compared to BB-12. Concerning Bacteroidata, previous studies have shown that bifidobacteria share a mutualistic relationship with *Bacteroides* spp. in the form of cross-feeding and the reestablishment of Bacteroides populations may be instrumental for restoring colonization resistance and a stable gut ecosystem.^6,15^ As the control group differed from the other groups at the baseline, the experiment might be repeated with an improved study design. An alternative would be to conduct a clinical trial and in doing so avoid animal experimentation in accordance with the 3 R principles (Replacement, Reduction, Refinement)^47^ as well as to avoid cross-species differences in colonization potential of bacterial species.

This study was a continuation to our previous study wherein we showed long-term colonization of donors´ bifidobacteria in rCDI patients in FMT context.^16^ Here, we showed that isolated strains can constitutively express gut colonization factors such as pilus genes under *in vitro* conditions and elicit a restorative effect in mice with antibiotic-induced dysbiosis. The strains´ properties are promising regarding their possible therapeutic use either as single strains or in combination with other gut commensals.

## Supplementary Material

Supplemental Material
