# Supplementary material for: In vitro adhesion, pilus expression, and in vivo amelioration of antibiotic-induced microbiota disturbance by Bifidobacterium spp. strains from fecal donors
Source: Gut Microbes. 2023 Jul 4;15(1):2229944. doi: 10.1080/19490976.2023.2229944 (PMC10321227; doi:10.1080/19490976.2023.2229944)
Supplement: Supplemental Material [file KGMI_A_2229944_SM9832.docx]

**Supplementary Material**

**Supplementary Table 1. Phenotypic identification of bacterial strains.**

| **SPECIES OR STRAIN** | **COLONY** | **GRAM STAINING** | **IDENTIFICATION** |
| --- | --- | --- | --- |
| **Strains administered or isolated during the mouse colonization trial** | | | |
| *Bifidobacterium animalis* subsp. *lactis* BB-12 | even edged | gram-positive short thick rod | MALDI-TOF (score: 2.28) |
| *Bifidobacterium longum* DX_pv18 | big slimy | gram-variable long curved rod | rep-PCR^1^ |
| *Bifidobacterium longum* DX_pv23 | medium slimy | gram-variable long curved rod | rep-PCR^1^ |
| *Bifidobacterium pseudolongum* | uneven pigmented | gram-variable amorphous rod | MALDI-TOF (score: >1.80^2^) |
| *Bifidobacterium pseudolongum* | slimy | gram-variable amorphous rod | MALDI-TOF (score: >1.80^2^) |
| *Ligilactobacillus murinus* | rugged | gram-positive stacked rod | MALDI-TOF (score: >1.80^2^) |
| *Ligilactobacillus murinus* | even pigmented | gram-positive stacked rod | MALDI-TOF (score: >1.80^2^) |
| *Ligilactobacillus murinus* | buttony | gram-positive stacked rod | MALDI-TOF (score: >1.80^2^) |
| **Strains used as MALDI-TOF MS reference** | | | |
| *Bifidobacterium adolescentis* DSM 20083 | nd | nd | MALDI-TOF (score: 2.05) |
| *Bifidobacterium angulatum* DSM 20098 | nd | nd | MALDI-TOF (score: 1.95) |
| *Bifidobacterium animalis* subsp. *lactis* DSM 10140 | nd | nd | MALDI-TOF (score: 2.22) |
| *Bifidobacterium bifidum* DSM 20456 | nd | nd | MALDI-TOF (score: 2.24) |
| *Bifidobacterium breve* DSM 20213 | nd | nd | MALDI-TOF (score: 2.29) |
| *Bifidobacterium catenulatum* DSM 16992 | nd | nd | MALDI-TOF (score: 2.12) |
| *Bifidobacterium dentium* DSM 20436 | nd | nd | MALDI-TOF (score: 2.16) |
| *Bifidobacterium gallicum* DSM 20093 | nd | nd | MALDI-TOF (score: 2.14) |
| *Bifidobacterium longum* subsp. *infantis* DSM 20088 | nd | nd | MALDI-TOF (score: 2.19) |
| *Bifidobacterium longum* subsp. *longum* DSM 20219 | nd | nd | MALDI-TOF (score: 1.99) |
| *Bifidobacterium pseudocatenulatum* DSM 20438 | nd | nd | MALDI-TOF (score: 2.15) |

^1^No peaks in MALDI-TOF. Identification by rep-PCR as described in Jouhten et al. (see ref. 16).

^2^Score based on multiple isolates.

nd = not done

**Supplementary Table 2. Pilus gene expression counts for *Bifidobacterium* spp. strains.^1^**

| **GENE** | **REPLICATE 1** | **REPLICATE 2** | **REPLICATE 3** |
| --- | --- | --- | --- |
| ***Bifidobacterium adolescentis* DX_pv1** | | | |
| Flp pilus assembly complex ATPase component TadA | 9,62 | 8,03 | 3,14 |
| pilus assembly protein | 2,14 | 0,89 | 0,00 |
| flp pilus-assembly TadE/G-like family protein | 3,21 | 0,00 | 0,00 |
| prepilin peptidase | 27,80 | 14,28 | 14,67 |
| ***Bifidobacterium pseudocatenulatum* DX_pv5** | | | |
| pilus assembly protein | 11,37 | 5,60 | 1,91 |
| SpaH/EbpB family LPXTG-anchored major pilin | 0,00 | 0,00 | 0,00 |
| prepilin peptidase | 4,55 | 8,27 | 0,96 |
| prepilin-type N-terminal cleavage/methylation domain-containing | 4,55 | 7,35 | 5,74 |
| prepilin-type N-terminal cleavage/methylation domain-containing | 12,51 | 29,41 | 6,70 |
| prepilin-type N-terminal cleavage/methylation domain-containing | 30,69 | 31,25 | 20,10 |
| type 4a pilus biogenesis protein PilO | 3,41 | 2,76 | 0,96 |
| Flp pilus assembly complex ATPase component TadA | 9,09 | ,8,27 | 9,57 |
| type IV pilus assembly protein PilM | 4,55 | 2,76 | 2,87 |
| flp pilus-assembly TadE/G-like family protein | 5,68 | 0,92 | 1,91 |
| PilT/PilU family type 4a pilus ATPase | 26,15 | 10,11 | 12,44 |

^1^Trimmed Mean of M-values was used as normalization method for counts in edgeR.


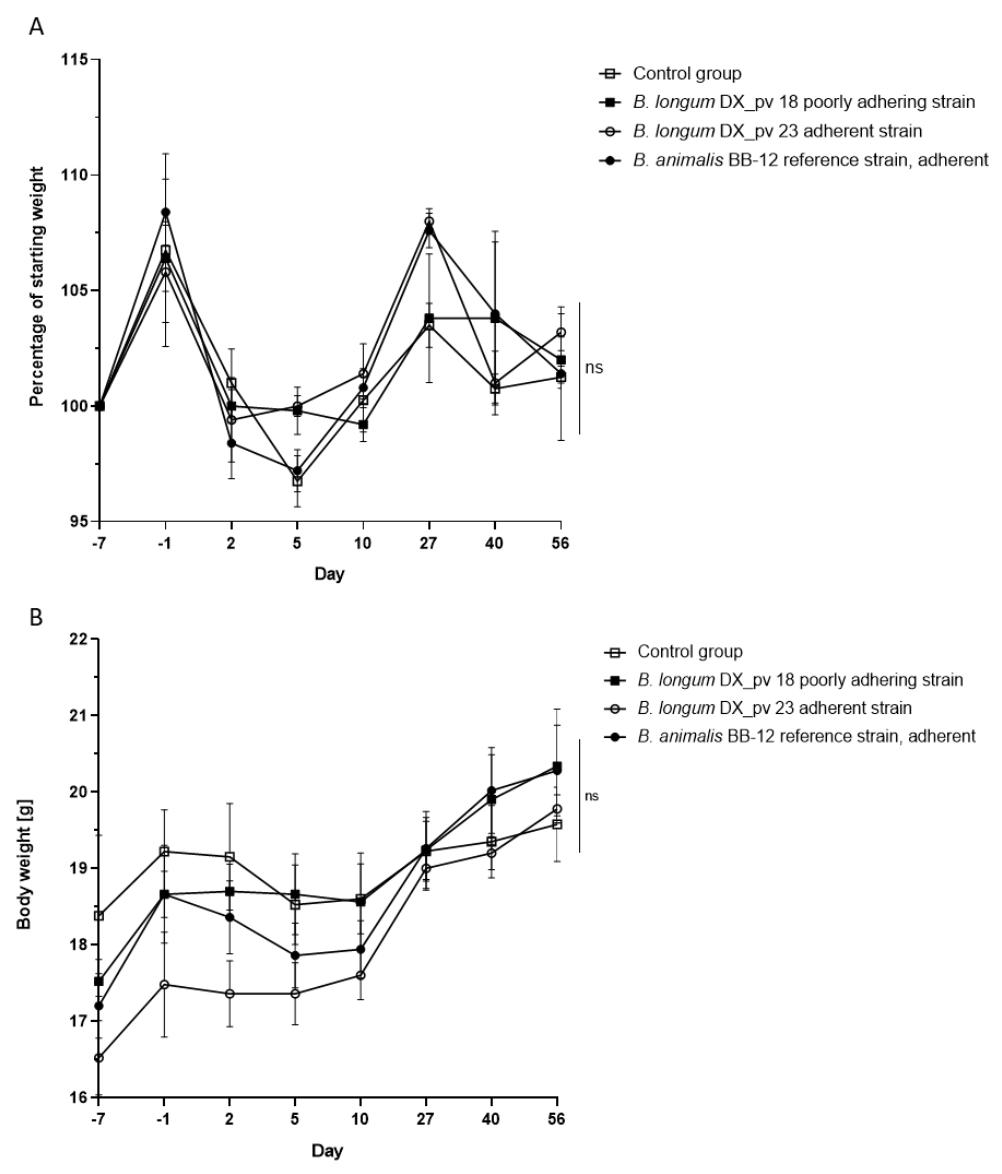
**Supplementary Figure 1. Changes in mouse body weight measured over the course of 56 days.** Body weight presented as individual weights of C57BL/6 mice (A). Body weight presented in relation to individual starting weight (B). Groups were compared using ANOVA followed by Tukey’s multiple comparisons test.


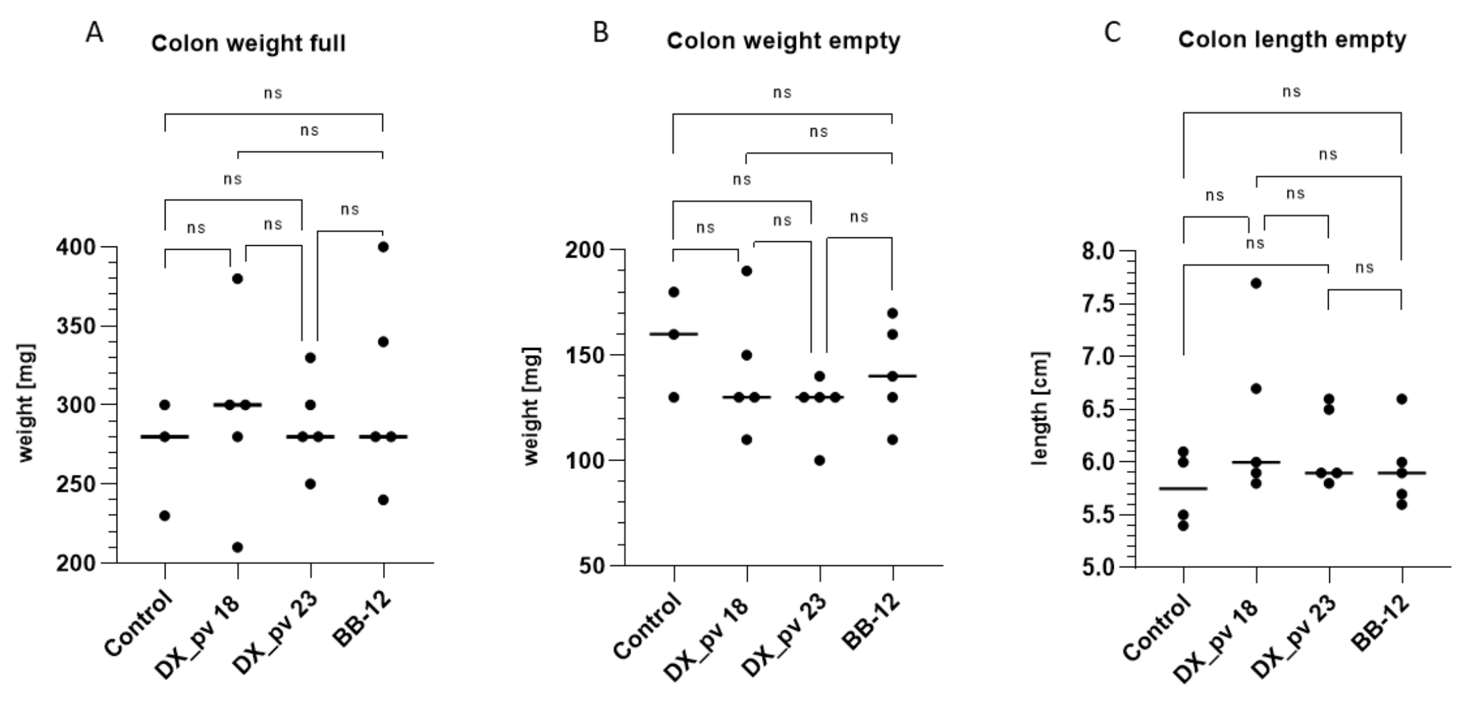
**Supplementary Figure 2. Mouse colon weight and length at necropsy (day 56).** Total colon weight before (A) or after (B) emptying stool, and the length of the emptied colon (C) of individual mice is provided for three treatment groups and control. Groups were compared using ANOVA followed by Tukey’s multiple comparisons test.


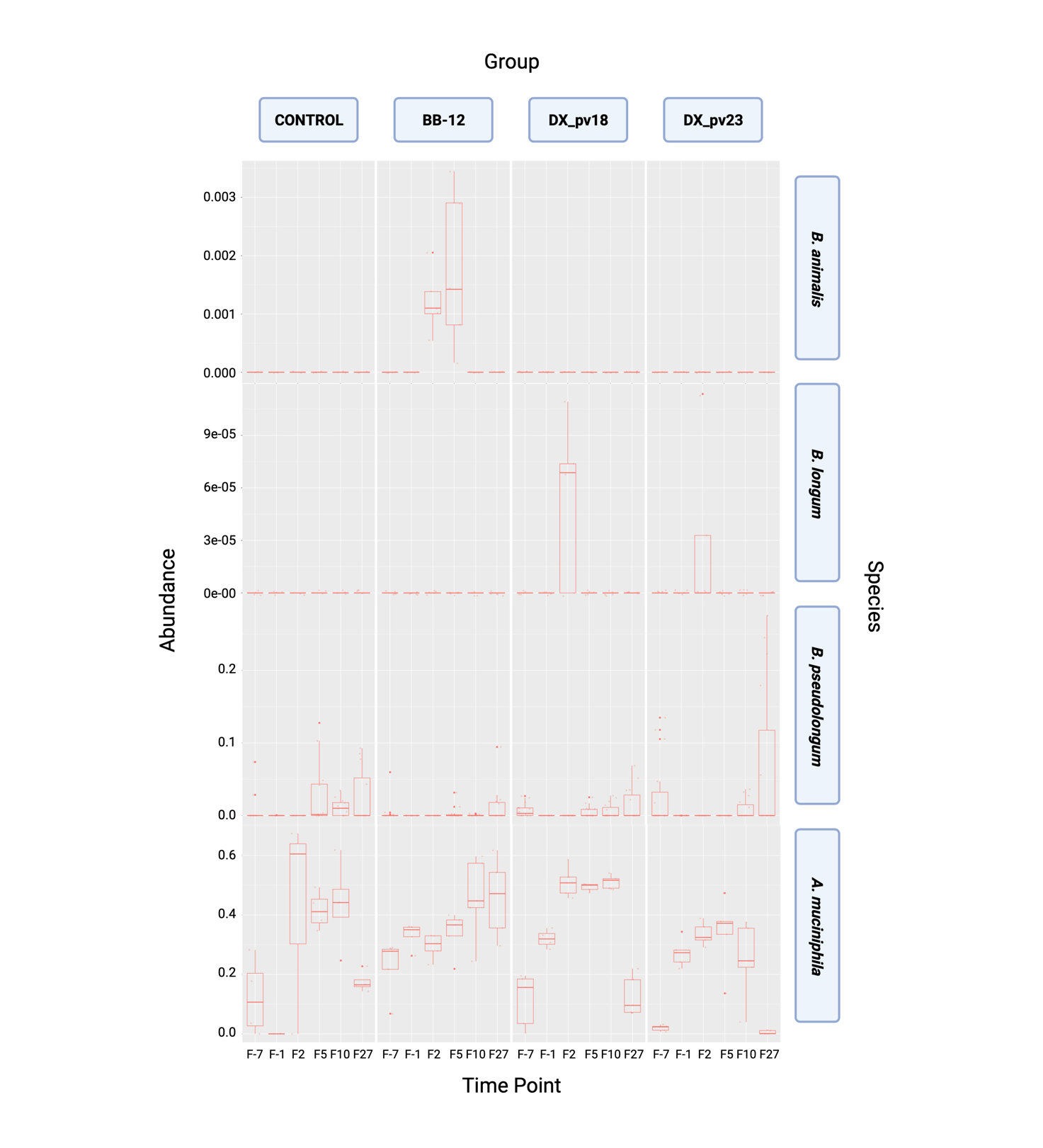
**Supplementary Figure 3. Presence of *Bifidobacterium* spp. and *Akkermansia muciniphila* in different treatment groups across time points.** Time points: F-7 and F-1: before and after antibiotic treatment, respectively; F2, F5, F10, F27: 2, 5, 10, and 27 days after administration.
